# Is the world of stroke research entering the Chinese era?

**DOI:** 10.3389/fneur.2023.1189760

**Published:** 2023-05-05

**Authors:** Wen-Jun Tu

**Affiliations:** ^1^Department of Neurosurgery, Beijing Tiantan Hospital of Capital Medical University, Beijing, China; ^2^Department of Radiobiology, Institute of Radiation Medicine, Chinese Academy of Medical Sciences & Peking Union Medical College, Tianjin, China

**Keywords:** stroke, research, China, population, clinical

Stroke is a major health concern globally, ranking as the second-leading cause of death and the third-leading cause of death and disability combined. The economic impact of stroke is also significant, with an estimated global cost exceeding US$721 billion, equivalent to 0.66% of the global GDP (1). In 2019, stroke accounted for 12.2 million new cases, 101 million prevalent cases, and 6.55 million deaths worldwide (2). In China, stroke is particularly prevalent, with 3.4 million new cases, 17.8 million prevalent cases, and 2.3 million deaths in 2020 alone (3). These figures are disproportionately high, considering that China accounts for only 18% of the world's population. In fact, China's stroke incidence and mortality rates are 28 and 35% respectively, higher than the global average. Furthermore, the estimated lifetime risk of stroke in China is 39.3% from the age of 25 years onwards, which is significantly higher than the global average of 24.9% (4).

This opinion article presents an analysis of the current state of stroke research in China, focusing on the four major clinical medicine journals, namely the New England Journal of Medicine (NEJM), The Lancet, the Journal of the American Medical Association (JAMA), and the British Medical Journal (BMJ). In addition, Frontiers in Neurology was also used as a reference. We also examine the factors driving progress in stroke research in China and discuss possible future directions for stroke research in the country.

China has made significant progress in stroke prevention, treatment, and rehabilitation in recent years (5, 6). The implementation of the stroke center model and stroke emergency map has greatly improved the acute care capacity (7, 8). Chinese researchers have also made remarkable contributions to the treatment of ischemic stroke through both endovascular thrombectomy and pharmacological approaches in the past year (9–13). For example, Huo et al. conducted a trial in China that demonstrated the superior outcomes of endovascular therapy within 24 h compared to medical management alone in patients with large cerebral infarctions (9). Another study found that thrombectomy was more effective in achieving good functional status at 90 days than medical therapy in patients with stroke caused by basilar-artery occlusion who presented 6 to 24 h after symptom onset (10). In a trial involving Chinese patients with basilar-artery occlusion, those who received both intravenous thrombolysis and endovascular thrombectomy had better functional outcomes at 90 days than those who received only best medical care (11). Regarding thrombolysis, Wang et al. showed that Tenecteplase was non-inferior to alteplase in individuals with ischemic stroke who were eligible for standard intravenous thrombolytic but ineligible for or refused endovascular thrombectomy (12). Moreover, Chen et al. found that remote ischemic conditioning significantly increased the likelihood of excellent neurologic function at 90 days compared to usual care among adults with acute moderate ischemic stroke (13).

As the population of China continues to age and risk factors for stroke, such as diabetes and hypertension, remain poorly controlled and on the rise, the incidence of stroke is reaching a period of high prevalence (5, 14). This presents a significant opportunity for clinical research, given the large number of patients affected. Furthermore, China's extensive university education system, implemented since the reform and opening up, has produced a substantial number of highly trained professionals in the field of stroke research. The emergence of influential figures in the stroke field, such as Wang Yongjun (who has published 28 articles in Frontiers in Neurology in the past 2 years), Zhao Xingquan (who has published 37 articles in Frontiers in Neurology in the past 2 years), and Ji Xunming, has significantly propelled the industry's development in China. As a result, Chinese stroke research is well-positioned to rapidly expand and potentially attain world leadership status.

It is important to note that stroke research in China has predominantly focused on ischemic stroke, with particular emphasis on acute-phase rescue and treatment during hospitalization. Societal organizations such as the Chinese Stroke Association and the National Health Commission Stroke Prevention and Control Committee have made significant efforts toward stroke prevention, post-discharge rehabilitation, and secondary prevention for the past decade (5, 15, 16). However, despite these efforts, scientific output has yet to receive significant academic recognition. Hemorrhagic strokes are the primary cause of death and disability resulting from stroke, despite minor strokes being predominant in China. According to a nationwide hospital-based cohort study, the in-hospital death rate for stroke inpatients in China ranged from 0.9% for ischemic stroke to 5.1% for intracerebral hemorrhage (17). In response, the Chinese government has launched the Healthy China 2030 plan and initiated “The Project to Reduce Millions of New Disabilities” with the aim of reducing the death and disability rates of patients with hemorrhagic stroke (18). Future research efforts should focus on finding effective rehabilitation programs for these patients, and government funding should be directed toward research on hemorrhagic strokes.

Our study focused on stroke-related research published between 2020 and 2021 in the Science Citation Index Expanded (SCIE) database. A total of 9,113 articles and review articles were identified using the search terms “stroke”. Of these, Chinese scholars contributed 2,351 publications, accounting for 25.8% of the total, and ranking second globally after the United States (2,720, 29.8%). Capital Medical University ranked first among research institutions worldwide with 428 publications. However, the average number of citations per article by Chinese scholars was only 90% of the global average (6.18/6.93), suggesting the need for further improvement in the quality of stroke-related research conducted by Chinese scholars.

As an example, Frontiers in Neurology published a total of 4,220 papers, including articles and reviews, between 2021 and 2022, according to the SCIE database. Of these, 673 (15.9%) were related to stroke research, with China contributing to nearly one-third of these papers (31.6%). Four highly representative articles (most cited articles in SCIE database) from these stroke-related studies include a review by Li et al. (19), which assessed the progress of hematoma expansion (HE) studies after intracerebral hemorrhage (ICH) in recent years. Chen et al. (20) investigated the individual and joint effects of Neutrophil-to-lymphocyte ratio and platelet-to-lymphocyte on functional outcomes of acute ischemic stroke (AIS), while Ren et al. (21) conducted a randomized clinical trial to assess whether conscious sedation is superior to general anesthesia for patients undergoing mechanical thrombectomy for AIS. Chen et al. (22) also elucidated the characteristics of wall shear stress and pressure of intracranial atherosclerosis analyzed by a computational fluid dynamics model. All four of these articles have been cited more than 20 times in the SCIE database as of March 13, 2023, with an average of 3.5 citations in the same period. Frontiers in Neurology remains open to stroke-related submissions, particularly from researchers based in China.

In conclusion, China has made significant strides in the field of stroke research, driven primarily by its economic growth, talent development, and demographic advantages. While there has been a notable increase in the number of studies published in top journals, the overall quality of research still falls behind that of developed countries. Moving forward, the government must invest more resources in stroke-related research, particularly in the areas of hemorrhagic stroke and rehabilitation. By doing so, Chinese scholars can make further contributions to the field of stroke research and improve the overall health of the global population.

## Author contributions

W-JT had full access to all the data in the study and took responsibility for the integrity of the data and the data analysis accuracy, study concept and design, acquisition of data, analysis and interpretation of data, drafting of the manuscript, critical revision of the manuscript for important intellectual content, and study supervision.
